# Supplementary material for: A Humanized Yeast Phenomic Model of Deoxycytidine Kinase to Predict Genetic Buffering of Nucleoside Analog Cytotoxicity
Source: Genes (Basel). 2019 Sep 30;10(10):770. doi: 10.3390/genes10100770 (PMC6826991; doi:10.3390/genes10100770)
Supplement: Supplementary file 1 [file genes-10-00770-s001.zip › Additional_Files/Additional_File7_GOTermHeatmaps.zip!/Additional_File7_GOTermHeatmaps/preribosome.pdf]

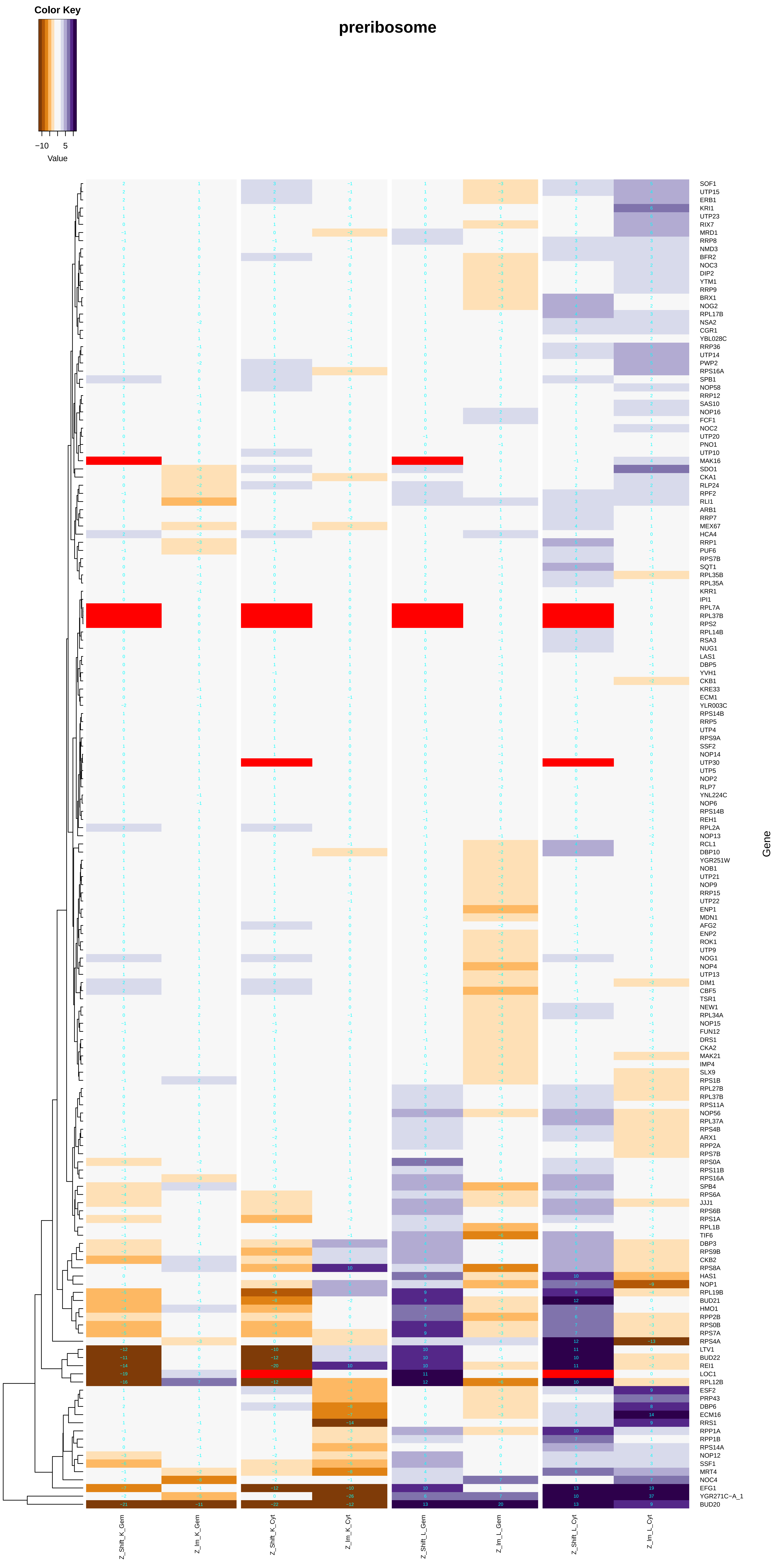

Color Key
−10
5
Value
preribosome
SOF1
UTP15
ERB1
KRI1
UTP23
RIX7
MRD1
RRP8
NMD3
BFR2
NOC3
DIP2
YTM1
RRP9
BRX1
NOG2
RPL17B
NSA2
CGR1
YBL028C
RRP36
UTP14
PWP2
RPS16A
SPB1
NOP58
RRP12
SAS10
NOP16
FCF1
NOC2
UTP20
PNO1
UTP10
MAK16
SDO1
CKA1
RLP24
RPF2
RLI1
ARB1
RRP7
MEX67
HCA4
RRP1
PUF6
RPS7B
SQT1
RPL35B
RPL35A
KRR1
IPI1
RPL7A
RPL37B
RPS2
RPL14B
RSA3
NUG1
LAS1
DBP5
YVH1
CKB1
KRE33
ECM1
YLR003C
RPS14B
RRP5
UTP4
RPS9A
SSF2
NOP14
UTP30
UTP5
NOP2
RLP7
YNL224C
NOP6
RPS14B
REH1
RPL2A
NOP13
RCL1
DBP10
YGR251W
NOB1
UTP21
NOP9
RRP15
UTP22
ENP1
MDN1
AFG2
ENP2
ROK1
UTP9
NOG1
NOP4
UTP13
DIM1
CBF5
TSR1
NEW1
RPL34A
NOP15
FUN12
DRS1
CKA2
MAK21
IMP4
SLX9
RPS1B
RPL27B
RPL37B
RPS11A
NOP56
RPL37A
RPS4B
ARX1
RPP2A
RPS7B
RPS0A
RPS11B
RPS16A
SPB4
RPS6A
JJJ1
RPS6B
RPS1A
RPL1B
TIF6
DBP3
RPS9B
CKB2
RPS8A
HAS1
NOP1
RPL19B
BUD21
HMO1
RPP2B
RPS0B
RPS7A
RPS4A
LTV1
BUD22
REI1
LOC1
RPL12B
ESF2
PRP43
DBP6
ECM16
RRS1
RPP1A
RPP1B
RPS14A
NOP12
SSF1
MRT4
NOC4
EFG1
YGR271C−A_1
BUD20
Gene
Z_Shift_K_Gem
Z_Im_K_Gem
Z_Shift_K_Cyt
Z_Im_K_Cyt
Z_Shift_L_Gem
Z_Im_L_Gem
Z_Shift_L_Cyt
Z_Im_L_Cyt

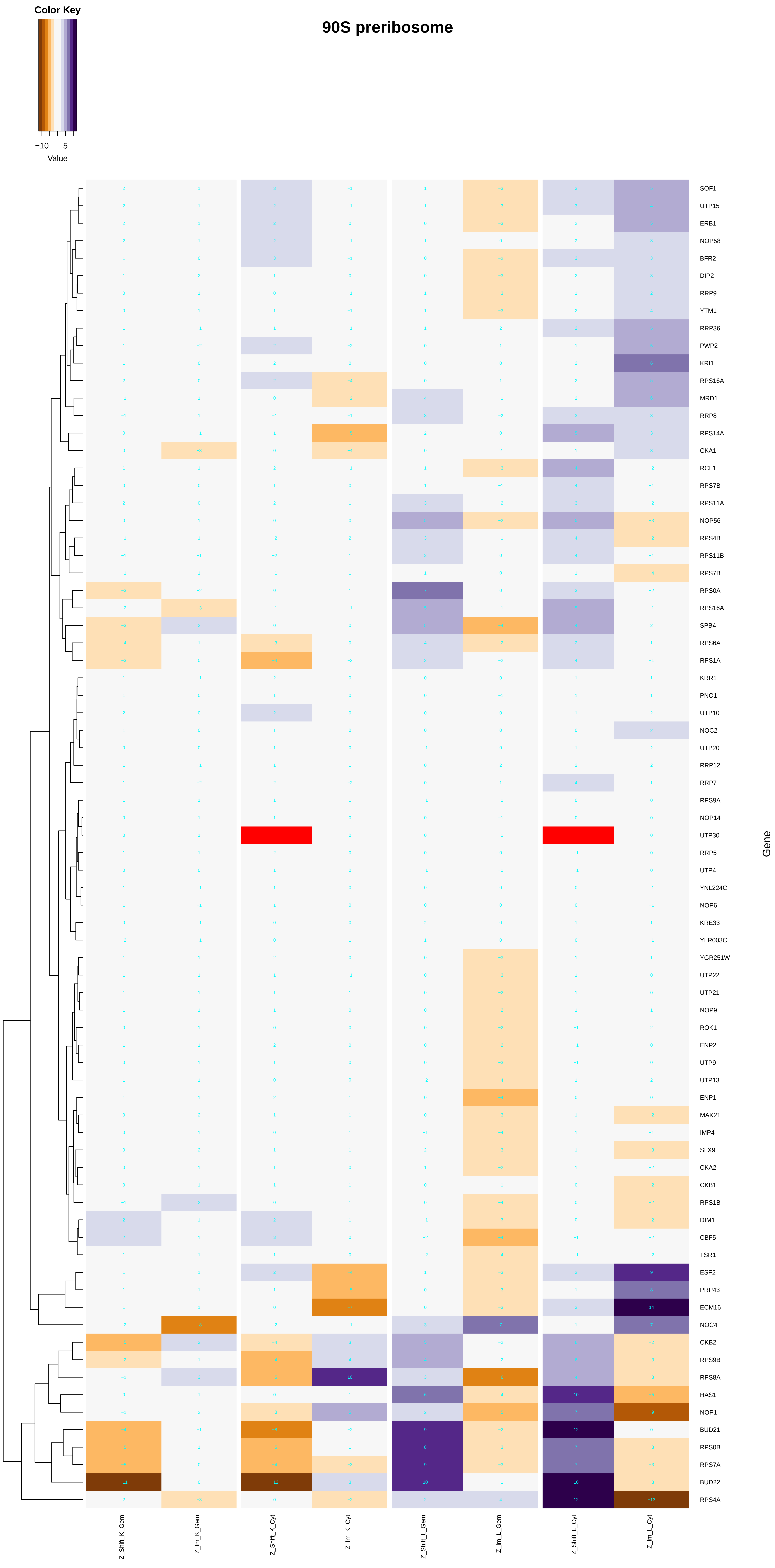
Color Key
−10
5
Value
90S preribosome
SOF1
UTP15
ERB1
NOP58
BFR2
DIP2
RRP9
YTM1
RRP36
PWP2
KRI1
RPS16A
MRD1
RRP8
RPS14A
CKA1
RCL1
RPS7B
RPS11A
NOP56
RPS4B
RPS11B
RPS7B
RPS0A
RPS16A
SPB4
RPS6A
RPS1A
KRR1
PNO1
UTP10
NOC2
UTP20
RRP12
RRP7
RPS9A
NOP14
UTP30
RRP5
UTP4
YNL224C
NOP6
KRE33
YLR003C
YGR251W
UTP22
UTP21
NOP9
ROK1
ENP2
UTP9
UTP13
ENP1
MAK21
IMP4
SLX9
CKA2
CKB1
RPS1B
DIM1
CBF5
TSR1
ESF2
PRP43
ECM16
NOC4
CKB2
RPS9B
RPS8A
HAS1
NOP1
BUD21
RPS0B
RPS7A
BUD22
RPS4A
Gene
Z_Shift_K_Gem
Z_Im_K_Gem
Z_Shift_K_Cyt
Z_Im_K_Cyt
Z_Shift_L_Gem
Z_Im_L_Gem
Z_Shift_L_Cyt
Z_Im_L_Cyt

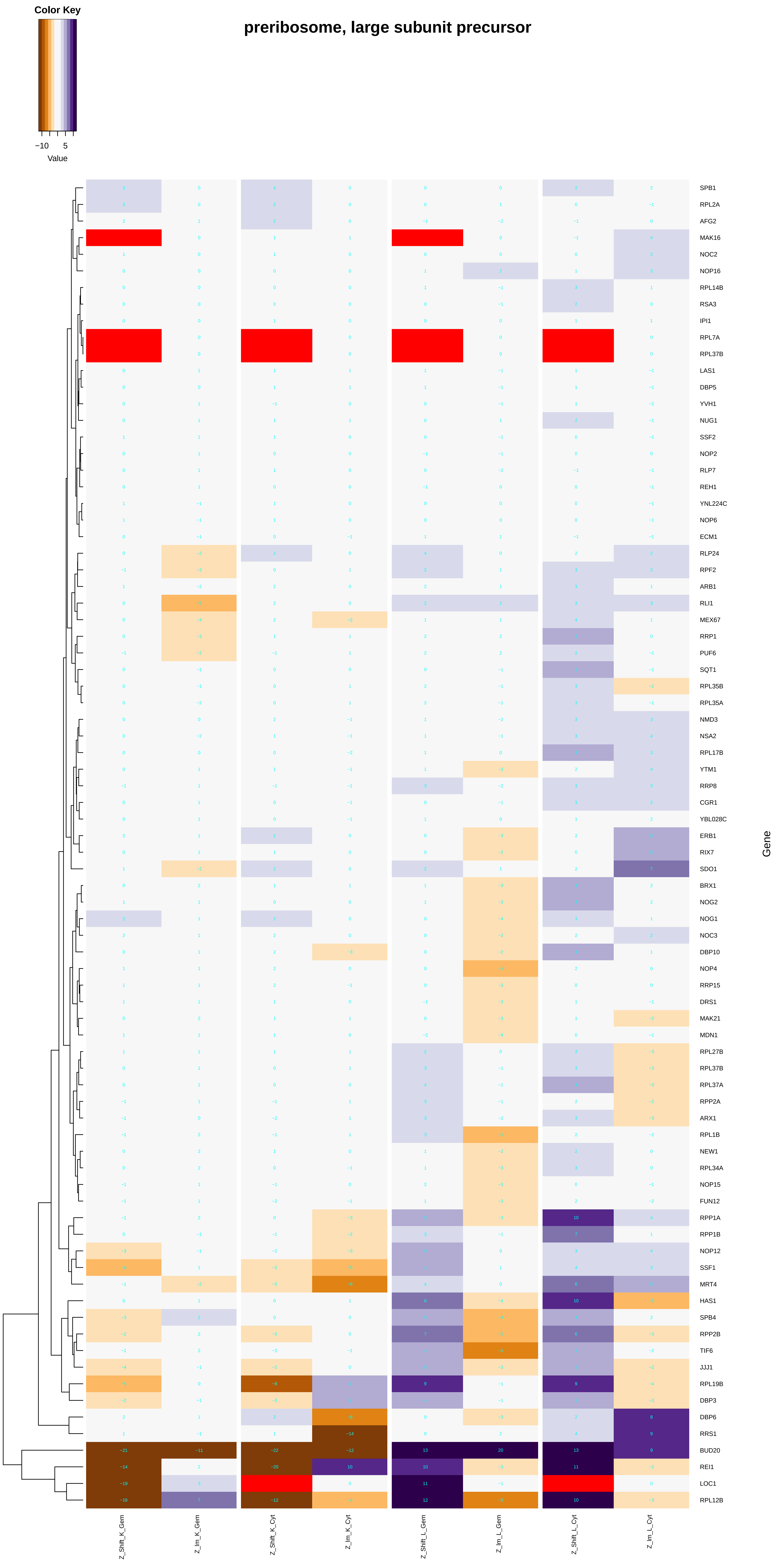
preribosome, large subunit precursor
Color Key
−10
5
Value
SPB1
RPL2A
AFG2
MAK16
NOC2
NOP16
RPL14B
RSA3
IPI1
RPL7A
RPL37B
LAS1
DBP5
YVH1
NUG1
SSF2
NOP2
RLP7
REH1
YNL224C
NOP6
ECM1
RLP24
RPF2
ARB1
RLI1
MEX67
RRP1
PUF6
SQT1
RPL35B
RPL35A
NMD3
NSA2
RPL17B
YTM1
RRP8
CGR1
YBL028C
ERB1
RIX7
SDO1
BRX1
NOG2
NOG1
NOC3
DBP10
NOP4
RRP15
DRS1
MAK21
MDN1
RPL27B
RPL37B
RPL37A
RPP2A
ARX1
RPL1B
NEW1
RPL34A
NOP15
FUN12
RPP1A
RPP1B
NOP12
SSF1
MRT4
HAS1
SPB4
RPP2B
TIF6
JJJ1
RPL19B
DBP3
DBP6
RRS1
BUD20
REI1
LOC1
RPL12B
Gene
Z_Shift_K_Gem
Z_lm_K_Gem
Z_Shift_K_Cyt
Z_lm_K_Cyt
Z_Shift_L_Gem
Z_lm_L_Gem
Z_Shift_L_Cyt
Z_lm_L_Cyt

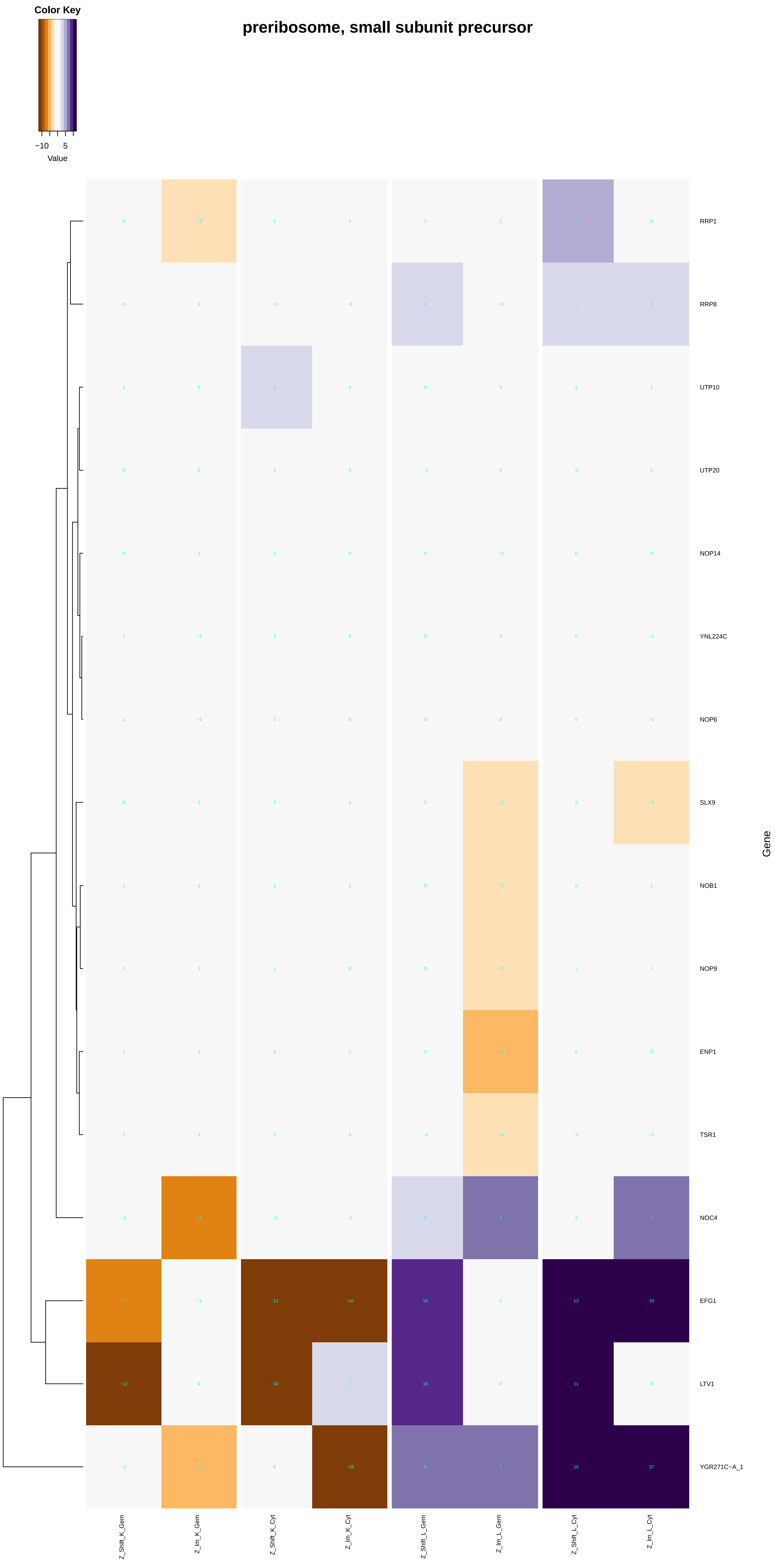

Color Key
−10
5
Value
preribosome, small subunit precursor
RRP1
RRP8
UTP10
UTP20
NOP14
YNL224C
NOP6
SLX9
NOB1
NOP9
ENP1
TSR1
NOC4
EFG1
LTV1
YGR271C−A_1
Gene
Z_Shift_K_Gem
Z_Im_K_Gem
Z_Shift_K_Cyt
Z_Im_K_Cyt
Z_Shift_L_Gem
Z_Im_L_Gem
Z_Shift_L_Cyt
Z_Im_L_Cyt

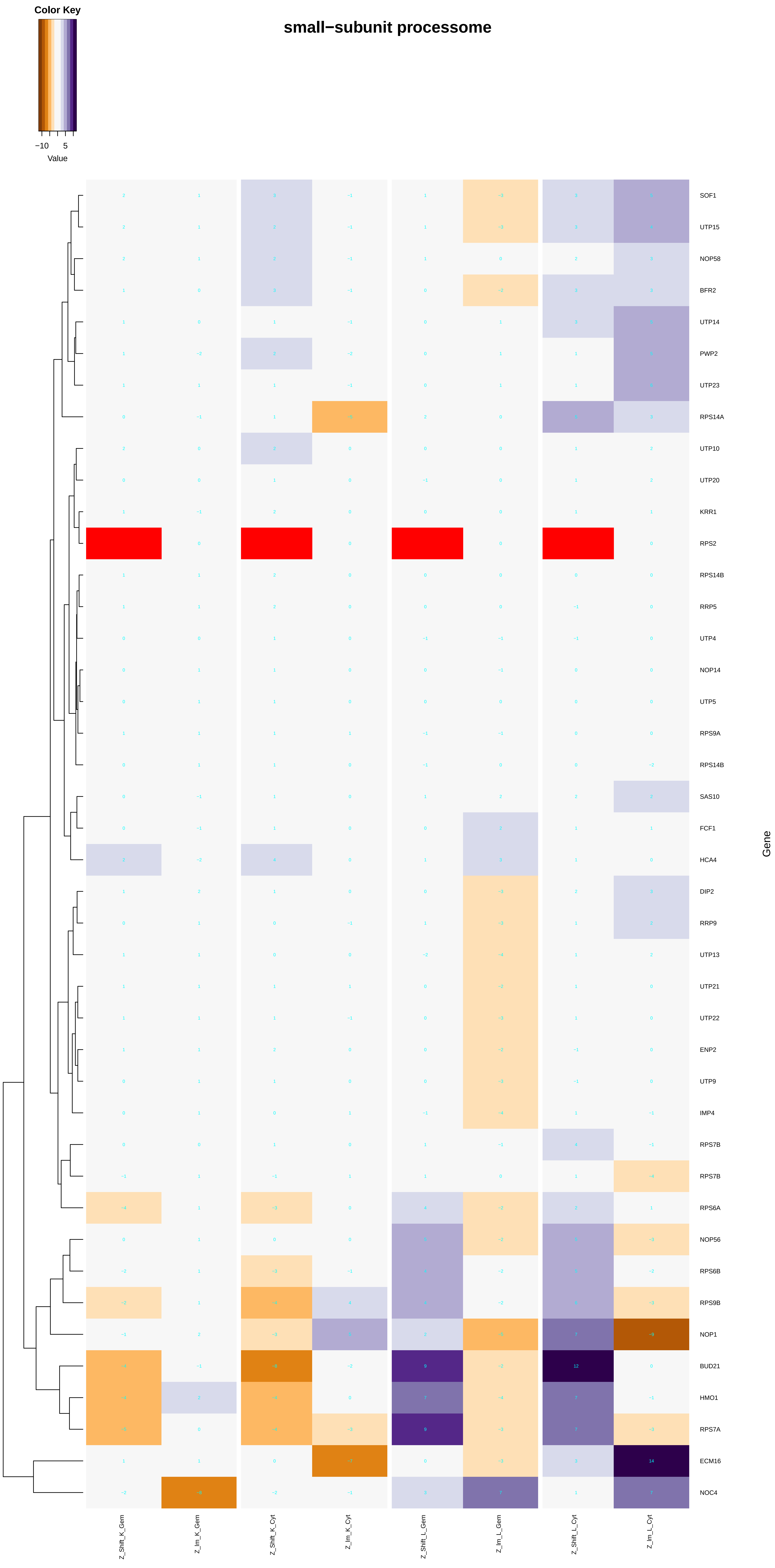
Color Key
−10 5
Value
small−subunit processome
SOF1
UTP15
NOP58
BFR2
UTP14
PWP2
UTP23
RPS14A
UTP10
UTP20
KRR1
RPS2
RPS14B
RRP5
UTP4
NOP14
UTP5
RPS9A
RPS14B
SAS10
FCF1
HCA4
DIP2
RRP9
UTP13
UTP21
UTP22
ENP2
UTP9
IMP4
RPS7B
RPS7B
RPS6A
NOP56
RPS6B
RPS9B
NOP1
BUD21
HMO1
RPS7A
ECM16
NOC4
Gene
Z_Shift_K_Gem
Z_Im_K_Gem
Z_Shift_K_Cyt
Z_Im_K_Cyt
Z_Shift_L_Gem
Z_Im_L_Gem
Z_Shift_L_Cyt
Z_Im_L_Cyt
